# Phylogenetic Characterization of β-Tubulins and Development of Pyrosequencing Assays for Benzimidazole Resistance in Cattle Nematodes

**DOI:** 10.1371/journal.pone.0070212

**Published:** 2013-08-12

**Authors:** Janina Demeler, Nina Krüger, Jürgen Krücken, Vera C. von der Heyden, Sabrina Ramünke, Ursula Küttler, Sandra Miltsch, Michael López Cepeda, Malcolm Knox, Jozef Vercruysse, Peter Geldhof, Achim Harder, Georg von Samson-Himmelstjerna

**Affiliations:** 1 Institute for Parasitology and Tropical Veterinary Medicine, Freie Universität Berlin, Berlin, Germany; 2 Niedersächsische Tierseuchenkasse, Hannover, Germany; 3 Institute for Parasitology, Hannover University of Veterinary Medicine, Hannover, Germany; 4 Dirección de Investigaciones (DE), Universidad Pedagogica y Tecnologica de Colombia (UPTC), Tunja, Boyacá, Colombia; 5 Commonwealth Scientific and Industrial Research Organisation (CSIRO) Animal, Food and Health Sciences, FD McMaster Laboratory, Chiswick, Armidale, New South Wales, Australia; 6 Department of Virology, Parasitology and Immunology, Faculty of Veterinary Medicine, Ghent University, Merelbeke, Belgium; 7 Institute for Biology, Heinrich-Heine-University Düsseldorf, Düsseldorf, Germany; Auburn University, United States of America

## Abstract

Control of helminth infections is a major task in livestock production to prevent health constraints and economic losses. However, resistance to established anthelmintic substances already impedes effective anthelmintic treatment in many regions worldwide. Thus, there is an obvious need for sensitive and reliable methods to assess the resistance status of at least the most important nematode populations. Several single nucleotide polymorphisms (SNPs) in the β-tubulin isotype 1 gene of various nematodes correlate with resistance to benzimidazoles (BZ), a major anthelmintic class. Here we describe the full-length β-tubulin isotype 1 and 2 and α-tubulin coding sequences of the cattle nematode *Ostertagia ostertagi*. Additionally, the *Cooperia oncophora* α-tubulin coding sequence was identified. Phylogenetic maximum-likelihood analysis revealed that both isotype 1 and 2 are orthologs to the *Caenorhabditis elegans* ben-1 gene which is also associated with BZ resistance upon mutation. In contrast, a *Trichuris trichiura* cDNA, postulated to be β-tubulin isotype 1 involved in BZ resistance in this human parasite, turned out to be closely related to *C. elegans* β-tubulins tbb-4 and mec-7 and would therefore represent the first non-ben-1-like β-tubulin to be under selection through treatment with BZs. A pyrosequencing assay was established to detect BZ resistance associated SNPs in β-tubulin isotype 1 codons 167, 198 and 200 of *C. oncophora* and *O. ostertagi*. PCR-fragments representing either of the two alleles were combined in defined ratios to evaluate the pyrosequencing assay. The correlation between the given and the measured allele frequencies of the respective SNPs was very high. Subsequently laboratory isolates and field populations with known resistance status were analyzed. With the exception of codon 167 in *Cooperia*, increases of resistance associated alleles were detected for all codons in at least one of the phenotypically resistant population. Pyrosequencing provides a fast, inexpensive and sensitive alternative to conventional resistance detection methods.

## Introduction

Benzimidazoles (BZs) represent a class of broad spectrum anthelmintic substances that have been widely used since the 1960's for helminth control in livestock and companion animals. This class includes several chemical compounds which all act by the same mode of action: the inhibition of polymerization of α- and β-tubulin monomers to microtubules. Thus, they interfere with the formation of the cytoskeleton, the mitotic spindle and the intracellular transport. The resulting disruption of the worm's metabolism is the detrimental factor of their anthelmintic effect. Within two or three days, inhibition of glucose uptake and intracellular transport leads to a lethal glycogen depletion of the worm [1]. Resistance against BZs was detected shortly after introduction of the first commercialized drug thiabendazole on the market [2]. Notably gastrointestinal nematodes of ruminants, in particular sheep, and horses are affected (for review see [3]). To date the spread of anthelmintic resistance causes most problems in the sheep industry and in horses. In general, it seems that in cattle parasites anthelmintic resistance emerges slower, however, recent evidence suggests this situation is changing [4], [5]. Many factors, such as treatment frequency, under-dosing or lack of refugia may contribute to the development of resistance, leading to a selection advantage of that part of the worm population which is able to tolerate the applied substances [6], [7]. The mechanism of BZ resistance has been studied in some detail during the past decades. In the late 1980's a single nucleotide polymorphism (SNP) within codon 200 of the *ben-1* β-tubulin gene was found to be associated with BZ resistance in the free-living nematode *Caenorhabditis elegans* [8]. The alteration from TTC to TAC causes expression of tyrosin instead of phenylalanin. The same SNP was described in *Haemonchus contortus* [9]. Via transformation of BZ susceptible *C. elegans* with *H. contortus* β-tubulin, containing the TAC- codon (tyrosin) at position 200, BZ resistance could be generated, while transformation of the TTC genotype did not change BZ susceptibility.

Several SNPs in β-tubulin genes of different organisms have been associated with BZ resistance [10]–[18], such as changes from GAA to GCA in codon 198 of *H. contortus* and from TTC to TAC in codon 167 of various nematode species. This diversity complicates the molecular detection of the nematode resistance status. While anthelmintic resistance appears to increase rapidly, regular surveys of drug efficacies on the farms are required to enable effective deworming programs [19]. A widely used *in vivo* method to detect resistance is the fecal egg count reduction test (FECRT) [20]. A further examination method, the egg hatch assay (EHA), determines the concentration of the anthelmintic which is required to inhibit hatching of 50% of nematode eggs *in vitro* [21], [22]. In particular the FECRT is labor and cost intensive and only provides reliable results if more than approximately 25% of the nematode population is resistant [20]. To date, several molecular tests have been described for some sheep, cattle and horse nematode species which are all based on the detection of resistance-associated SNPs of the β-tubulin gene. Here, conventional PCR [23]–[25], real-time PCR [26]–[28] as well as pyrosequencing [29], [30] technologies have been employed. The latter two approaches principally allow the evaluation of SNP frequencies in DNA extracted from pooled parasites reducing efforts and costs thus making them amenable for use in routine field diagnosis. In the present study, we amplified the α-tubulin coding sequences of the gastrointestinal cattle nematodes *Cooperia oncophora* and *Ostertagia ostertagi* as well as of the β-tubulin isotype 1 and 2 of *O. ostertagi*. Furthermore, we describe a pyrosequencing assay to determine allele frequencies and genotypes in the BZ-resistance associated codons 167, 198 and 200 of the β-tubulin isotype 1 gene of *C. oncophora* and *O. ostertagi*. Due to the initial lack of available naturally resistant *C. oncophora* and *O. ostertagi* populations, DNA fragments containing the respective SNPs variants were initially generated artificially via *in vitro* mutagenesis. Thus, we were able to test different sets of SNP combinations in the codons 167, 198 and 200 over a wide range of predefined allele frequencies and to provide a practical tool to determine the resistance status of field populations of two important gastrointestinal cattle nematodes. Finally, we obtained and examined resistant field populations from Argentina, Australia, Columbia and Germany for the presence of these SNPs.

## Materials and Methods

### Ethics Statement

All animal experiments were performed in strict accordance to the German law for animal welfare (Tierschutzgesetz) and with the approval of the respective local authority. The studies performed in Germany were authorized by 1) Niedersächsisches Landesamt für Verbraucherschutz und Lebensmittelsicherheit (LAVES) under the reference numbers 509c42502-01A48 and 509.6-42502/3-04/872 and 2) Landesamt für Gesundheit und Soziales (LAGeSo), Berlin, under the reference number L0088/10. The experimental study performed in Colombia was locally authorized by the Comité de Ètica de la Investigación of the Universidad Pedagógica y Tecnológica de Colombia under the reference number ACTA01.

In Germany the calves (between 10–14 weeks of age) were kept indoors on straw and fed with hey *ad libitum* and pellets.

### Recovery of adult worms for RNA isolation

Helminth-free calves (treated with 7.5 mg/kg live weight albendazole 10 days prior to infection) were infected orally with 10,000 third stage larvae (L3) of either *C. oncophora* or *O. ostertagi* on three consecutive days (30,000 L3 in total). Susceptible *C. oncophora* (*C.o.*sus.) and *O. ostertagi* (*O.o.*sus.), both initially obtained from the Central Veterinary Laboratory at Weybridge, UK, and further passaged without anthelmintic challenge, were used for mono specific experimental infections.

The infected calves were euthanized by bleeding through opening the carotid artery immediately following captive bolt stunning, worms were collected directly from the small intestine or abomasum, respectively, and stored temporarily in 0.9% NaCl. Afterwards worms were examined microscopically to confirm species and sex. Collected worms were washed and stored at −80°C in diethyl pyrocarbonate (DEPC) treated water.

### Cooperia oncophora and Ostertagia ostertagi laboratory isolates

Helminth-free calves (treated with 88.5 mg/kg live weight levamisole 14 days prior to infection) were orally infected with 35,000 L3 of either *C. oncophora* or *O. ostertagi*. Susceptible *C. oncophora* (*C.o.*sus.) and *O. ostertagi* (*O.o.*sus.), both initially obtained from the Central Veterinary Laboratory at Weybridge, UK, and an in-house BZ-selected isolate of *O. ostertagi* (*O.o.*BZ-sel., currently tolerating 40% of the recommended therapeutic dose of albendazole) originating from the susceptible Weybridge isolate were used for mono specific experimental infections.

Feces from the infected calves were collected and submitted to fecal cultures (using fine chipped wood, 25°C, 75% humidity for 6–8 days). L3 were recovered using a Baerman funnel system and stored in ventilated cell culture flasks at 10°C for further use.

Species specific PCR was used in order to detect any possible contamination of larval cultures with other trichostrongylid species.

### Cooperia oncophora and Ostertagia ostertagi field populations

Larval cultures derived from naturally infected animals in the field with hints of BZ resistance (named *O.o.*Hamilton2010) were sent from Australia (original isolate obtained from Dr. David Rendell during a follow up study to [31]) to Germany in order to infect calves and determine the species present in the population. Infection of calves and recovery of L3 were performed as described above. Semi-qualitative real time PCR revealed >95% *Ostertagia* and <5% *Cooperia*.

A second field population was obtained from naturally infected calves from an organic farm in Schleswig-Holstein, North Germany. Animals were still excreting strongylid eggs following treatment with ivermectin (0.2 mg/kg bodyweight, subcutaneously, Ivomec®, Merial) and albendazole (7.5 mg/kg bodyweight, orally, Valbazen®, Pfizer). FECR data for animals treated with IVM was obtained on day 14, for animals treated with albendazole on day 7. Fecal samples were collected, cultured as described above and the obtained larvae were passaged under selection pressure of both, ivermectin and albendazole in therapeutic dosages. PCR analysis revealed *Cooperia* and *Ostertagia* and it was named Ger-BZ.

Larval cultures from Columbia were obtained from animals on a University Research farm (in Paipa), naturally infected with gastro-intestinal nematodes. Animals were randomly allocated into two treatment groups and a FECRT was performed as described for the Ger-BZ population. Therefore, larvae were derived from calves after treatment with the recommended dose of (A) 0,2 mg ivermectin/kg bodyweight (subcutaneously, Ivomec®, Merial), named Col-ML or (B) 7,5 mg albendazole bodyweight (orally, Vetanco, Argentina), named Col-BZ. Larvae were send to Germany and were used for DNA isolation without further passage in animals.

A further set of larval cultures were obtained in Argentina from naturally infected calves after treatment with the recommended dose of (A) ivermectin (0.2 mg/kg, Ivomec®), named Arg-ML or (B) fenbendazol (7.5 mg/kg, MSD Panacur®), named Arg-BZ (FECRT were performed at EEA INTA, Instituto Nacional de Tecnología Agropecuaria, Rafaela, Santa Fe). DNA was isolated in Argentina and send to Germany where it was used subsequently for species identification and in pyrosequencing analyses. FECRT were performed as described above.

FECRT data were calculated using the BootStreat program [32]. Reduced efficacy was assumed when FECR was <95% and the lower limit of the 95% confidence interval was <90%.

### Identification of coding sequences of α-tubulin, β-tubulin isotype 1 and 2

RNA was isolated from batches of 10–40 adult *C. oncophora* or *O. ostertagi*, respectively. Worms were homogenized mechanically using mortar, pestle and liquid nitrogen. The homogenized tissue was transferred into a guanidinium-thiocyanate buffer provided by the QuickPrep™Micro mRNA Purification Kit (Amersham Biosciences, Freiburg, Germany). The following mRNA-Isolation was carried out according to the manufacturer's protocol. cDNA synthesis was done using BD SMART™ RACE cDNA Amplification Kit (Clontech, St-Germain-en-Laye, France).

For amplification of β-tubulin isotype 1 fragments of *O. ostertagi*, degenerated primers previously described by Pape [33], were used. A fragment of *O. ostertagi* β-tubulin isotype 2 was available in-house from previous work. Rapid Amplification of cDNA Ends (RACE) was carried out for completion of coding sequences using gene specific primers. They were combined with either a primer complementary to the spliced leader 1 sequence to generate a fragment upstream including the 5′ UTR or the universal 3′ primer provided within the RACE Kit. A fragment of *O. ostertagi* α-tubulin was identified by chance during the β-tubulin isotype 2 RACE experiments and the sequence was then completed as described above. The α-tubulin-RACE primers for *O. ostertagi* were successfully employed for *C. oncophora* as well. Full length PCR products of *O. ostertagi* β-tubulin isotype 1, isotype 2 and α-tubulin and *C. oncophora* α-tubulin were obtained, cloned (TOPO® TA Cloning Kit, Invitrogen, Karlsruhe, Germany) and sequenced (SeqLab Laboratories, Göttingen, Germany).

### Phylogenetic analyses

The accession numbers of all cDNA sequences used are given in Table S1. Untranslated regions were removed from sequences and open reading frames were aligned using Clone Manager 9 Professional Edition (Scientific & Educational Software, Cary, NC) using exhaustive pairwise alignments of all sequences and progressive assembly by neighbor joining. Sequences were aligned as proteins using the BLOSSUM 62 similarity matrix but displayed and exported to interleaved Phylip format as aligned codons. Best substitution models were estimated by maximum likelihood calculations using jmodeltest 0.1.1 [34]. The model performing best under both the Akaike and Bayesian information criteria was used for calculating phylogenetic trees. Maximum likelihood phylogenetic tree for proteins was then calculated with PhyML 3.0.1 [35] using both, the Shimodaira-Hasegawa [SH] modification of the approximate likelihood test and the Bayesian transformation of the approximate likelihood ratio test option. The TIM2+G was identified by jmodeltest as the optimal nucleic acid substitution model and applied to both calculation methods. The proportion of invariable sites was set to 0 and the number of substitution rate categories modeling variation of evolution rate between sites was set to eight. The Γ distribution parameter for substitution rate categories was set to 0.526 as calculated by jmodeltest. PhyML was used to estimate base frequency equilibriums. Substitution rates R in the TIM2 model were set according to the jmodeltest results with R(A→C) = R(A→T) = 1.4533, R(C→G) = R(G→T) = 1, R(A→G) = 3.057, and R(C→T) = 6.0055. Five random trees and one tree obtained from the neighbor-joining BIONJ algorithm implemented in PhyML were used as starting points to avoid that the approximation might get trapped in a local maximum of the likelihood function. For optimization of tree topology the best of nearest neighbor interchange (NNI) and subtree pruning and regraftment (SPR) moves was chosen. The resulting tree with the highest likelihood in Newick format was visualized and processed using MEGA5 [36].

### Development of a pyrosequencing assay for genotyping and determination of β-tubulin allele frequencies

A pyrosequencing assay for the quantitative analysis of BZ resistance associated SNPs in the codons 167, 198 and 200 of the β-tubulin genes of *C. oncophora* and *O. ostertagi* was developed. Since natural resistance alleles in the β-tubulin were not available at the start of the project, several fragments with artificially inserted combination of SNPs in the codons 167, 198 and 200 were generated by fusion PCR. Using primers with mismatches at these positions, two fragments of β-tubulin for each species were amplified containing either the susceptible or the resistance associated allele at the respective codon. Genomic DNA of *C. oncophora* and *O. ostertagi* isolates was used as template. Primers are listed in Table S2. PCR was carried out in 25 µl using 5 U HotFire polymerase (Solis Biodyne, Tartu, Estonia), 1.5 mM MgCl_2_, 80 µM dNTPs, 200 nM specific primers. Cycling conditions for all initial PCRs were as follows: Denaturation at 95°C for 15 min, one cycle of 94°C 1 for min, 50°C for 2 min and 72°C for 1 min, followed by 39 cycles of 94°C 1 for min, 50°C for 1 min and 72°C for 1 min, followed by 72°C for 10 min as final elongation. The resulting PCR products Coβ-tub167s and Coβ-tub167re as well as Ooβ-tub167s and Ooβ-tub167re (see Table S2) were purified from agarose gels, mixed equally and used as template for the following fusion PCR. Primer pairs for this PCR were Co167PCRfw/CoPCR198/200rev and Oo167PCRfw/OoPCR198/200rev, respectively. Subsequently, PCR products were cloned using the pCR4 TOPO Vector, transformed into Top 10 Cells (TOPO® TA Cloning Kit, Invitrogen, Karlsruhe, Germany) and finally plasmid DNA was prepared using the NucleoSpin miniprep kit (Macherey and Nagel, Düren, Germany). Clones with mutagenized codons 167, 198, and 200 were identified by sequence analysis (GATC Biotech, Konstanz, Germany) and those containing the complete PCR products were used for further experiments.

For pyrosequencing assays, plasmid DNA (50 ng/µl) of β-tubulin fragments of *C. oncophora* and *O. ostertagi* containing either the susceptible or resistance associated allele in codon 167 or 198 and 200 were mixed in 11 different ratios to obtain different percentages of the BZ resistance associated alleles. In the following PCRs, these mixtures were used as templates. PCR was carried out in 25 µl using 5 U HotFire polymerase (Solis Biodyne, Tartu, Estonia), 1.5 mM MgCl_2_, 80 µM dNTPs, 200 nM specific primers, with the reverse primers carrying a biotin tag, and 1 µl of DNA template. Primer sequences are listed in Table S3. Cycling conditions were as follows: 15 min at 95°C polymerase activation step followed by 40 cycles with 95°C for 1 min, 53°C for 1 min and 72°C for 1 min, and a final elongation step at 72°C for 10 min. For the subsequent pyrosequencing assays, specific sequencing primers were used (Table S3). The pyrosequencing assays were performed using the PSQ™ MA-96A (Biotage, Hamburg, Germany) instrument and the PSQ™ SNP Reagents Kit according to the manufacturer's protocols. Each assay was performed in two independent experiments. Pearson correlations with 95% confidence bands were calculated using SigmaPlot11.

After establishment of the pyrosequencing assay the workgroup moved to Berlin. In order to improve accuracy PCRs were carried out using Phusion® polymerase (Thermo Scientific, St. Leon-Rot, Germany) which has strong proof-reading activity. Accordingly the PCR protocol had to be modified and was performed as follows: In 50 µl 1×Phusion HF, 0.2 mM dNTPs (Thermo Scientific), 250 nM of each primer, 10 µl Q solution, 5 µl CoralLoad (Qiagen), 1 µl Phusion II enzyme and 5–50 ng genomic DNA. After initial denaturation at 98°C for 30 s, forty cycles with 98°C for 10 s, a primer pair specific annealing temperature for 30 s and elongation at 72°C for 30 s followed by a final elongation at 72°C for 10 min was performed. Analysis of the field populations were performed on the PyroMark Q24 instrument using the PyroMark Gold Q24 reagents (Qiagen, Hilden, Germany). Dispersion patterns for all assays are shown in Table S4.

For *Haemonchus* spp. a previously published assay [30] for *H. contortus* β-tubulin-1 SNP detection was conducted on the new instrument PyroMark Q24 using the PyroMark Q24 reagents and the same PCR protocol with Phusion polymerase as described above. A second SNP was used to determine the amount of *H. contortus* and *Haemonchus placei* in the samples.

First, allele frequencies were compared to the susceptible reference isolate using a one-way ANOVA followed by Dunnett's multi comparison post hoc test. In a second analysis, differences in allele frequencies after treatment of the parental population with ML or BZ were analyzed using a Student's t test.

## Results

### Tubulin sequences of *C. oncophora* and *O. ostertagi*

We identified the complete coding sequences of *C. oncophora* (*Con*tba-1, Genbank Acc. No. JF930342) and *O. ostertagi* α-tubulin (*Oos*tba-1, Genbank Acc. No. JF930343) as well as β-tubulin isotype 1 (*Oos*tbb-1, Genbank Acc. No. JF930344) and β-tubulin isotype 2 (*Oos*tbb-2, Genbank Acc. No. JF930345) of *O. ostertagi*. The complete coding sequences of the *C. oncophora* and *O. ostertagi* α-tubulin are both 1338 bp in length (446 amino acids). The GC-content is approximately 48%, the molecular weight of the deduced protein is 50 kDa and the isoelectric point 4.76. The cDNA-sequences show 99% identity to each other. The complete coding sequence of *O. ostertagi* β-tubulin-1 is 1344 bp in length (corresponding to 448 amino acids), the GC-content is 48.1%, the calculated molecular weight of the protein is 49.9 kDa and its isoelectric point is 4.65. The identity of the cDNA-sequence to *C. oncophora* β-tubulin-1 (Genbank Acc. No. AY259994) is 99%. The *O. ostertagi* coding sequence of β-tubulin-2 is 1344 bp in length (448 amino acids), the GC-content is 45.3%, the calculated molecular weight for the deduced protein is 49.8 kDa and the isoelectric point 4.61.

### Phylogenetic analysis

**Figure 1:**
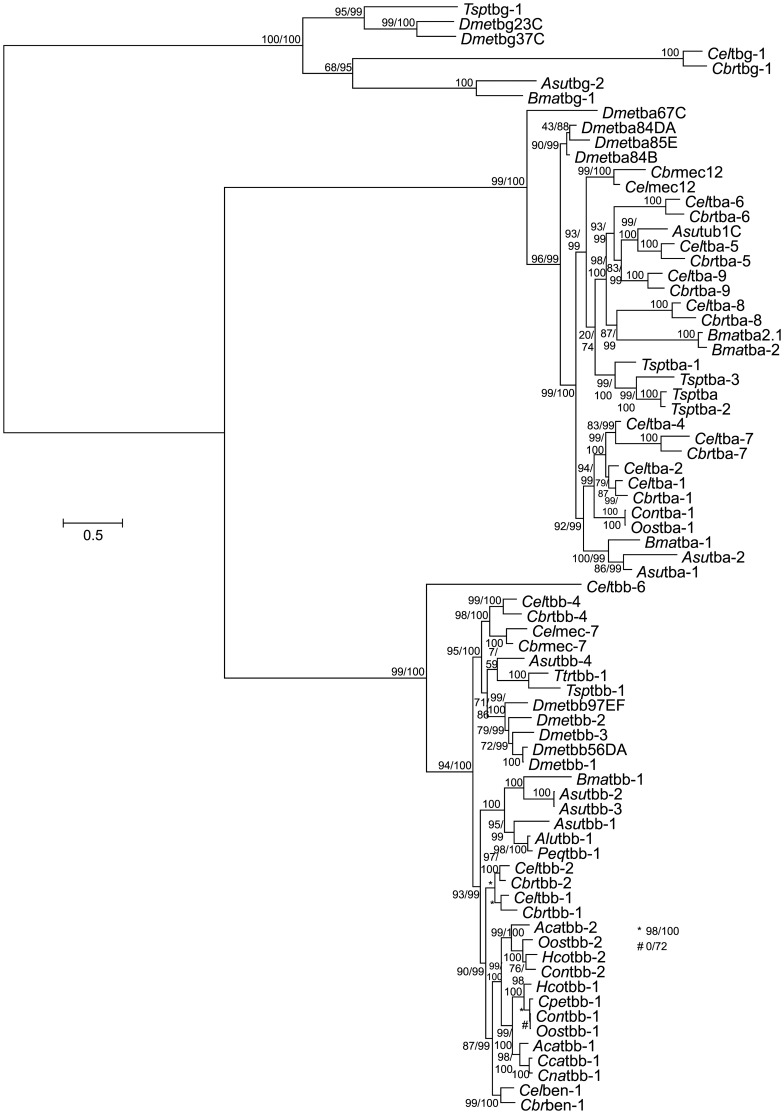
Phylogram showing relationship between nematode and *D. melanogaster* tubulins. Deduced proteins were aligned and the corresponding alignment of cDNAs with aligned codons was analyzed by maximum likelihood analysis as described in material and methods. Statistical support according to the Shimodaira-Hasegawa modification of the approximate likelihood test and the Bayesian transformation of the approximate likelihood ratio are reported before and after the slash. If only one number is given the results of both tests were identical. Accession numbers for all cDNAs are provided in Table S2. Species abbreviations: *Aca*, *Ancylostoma caninum*; *Alu*, *Ascaris lumbricoides*; *Asu*, *Ascaris suum*; *Bma*, *Brugia malayi*; *Can*, *Cylicocylus nassatus*; *Cbr*, *Caenorhabditis briggsae*; *Cca*, *Cylicocyclus catenatus*; *Cel*, *Caenorhabditis elegans*; *Con*, *Cooperia oncophora*; *Cpe*, *Cooperia pectinata*; *Dme*, *Drosophila melanogaster*; *Hco*, *Haemonchus contortus*; *Oos*, *Ostertagia ostertagia*; *Peq*, *Parascaris equorum*; *Tsp*, *Trichinella spiralis*; *Ttr*, *Trichuris trichiura*. An enlarged version of the β-tubulin subtree is provided in Figure S1.

The cDNA sequences were compared to other nematode and non-nematode (*Drosophila melanogaster*) tubulins and phylogeny was calculated using maximum likelihood and the TIM2 model for nucleotide substitution with Γ shape parameter. The resulting phylogram (Figure 1 and partly enlarged in Figure S1) shows a classification of all three tubulin families, α- β-, and γ-tubulins. The identified tubulins of *C. oncophora* and *O. ostertagi* are assigned to the correct major tubulin groups. All strongylid β-tubulin isotype 1 and 2 proteins are grouped together with a ≥99% statistical support. This branch significantly (87% and 99% with the two variants of the approximate likelihood test) clusters together with the *Caenorhabditis* ben-1 cDNAs, loss of which causes BZ resistance in *C. elegans*. The *Caenorhabditis* β-tubulins of isotype 1 and isotype 2 form a sister operational taxonomic unit to this branch. Obviously, the terms isotype 1 and isotype 2, as coined independently for *Caenorhabditis* and strongylids, do not refer to orthologs and do not designate phylogenetically meaningful β-tubulin families, but are only useful within the individual phylogenetic groups. Moreover, it must be stated that the previously described β-tubulin isotype 1 (*Alu*Tbb-1) sequence of *Ascaris lumbricoides* [18] does not belong to the same phylogenetic cluster as the *Caenorhabditis* and the strongylid β-tubulin isotype 1 and isotype 2 sequences. Instead, it forms an independent group together with a *P. equorum* β-tubulin cloned in our laboratory, three β-tubulins obtained from the *A. suum* transcriptome data recently deposited in GenBank and a *Brugia malayi* β-tubulin. The *T. trichiura* β-tubulin-1 recently described by Diawara [18] was found to be closely related to a *Trichinella spiralis* tbb-1 and an *A. suum* tbb-4. This whole branch, however, turned out not to be closely related to any of the rhabditid tbb-1, tbb-2 or ben-1 cDNAs but to the *D. melanogaster* β-tubulins and *Caenorhabditis* spp. tbb-4 and mec-7. Overall, these results indicate that diversification of β-tubulins by gene duplications occurred several times independently in different nematode lineages thus complicating a useful nomenclature.

### Pyrosequencing assay evaluation

**Figure 2:**
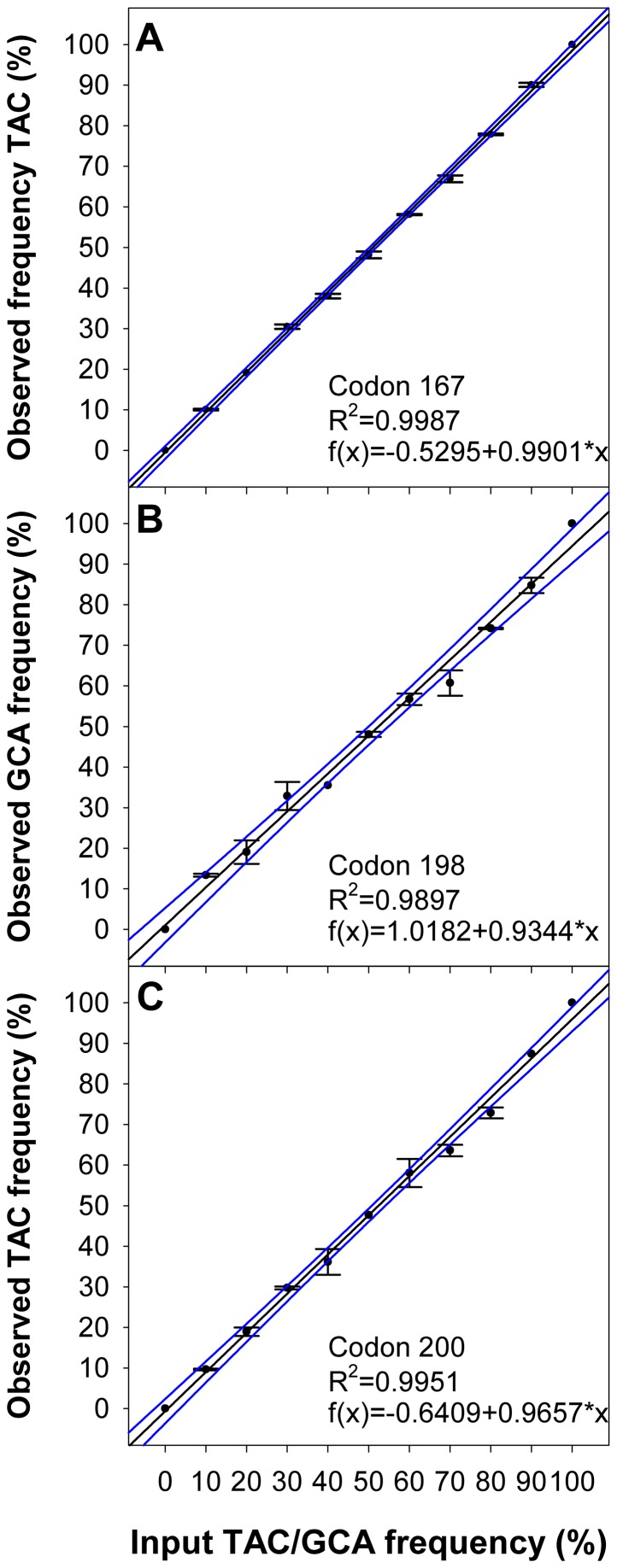
Regression analysis for pyrosequencing assays of *C. oncophora* β-tubulin isotype 1. Artificial mixtures of respective PCR-products as templates containing varying amounts of copies with TAC in codon 168 (A), codon 198 (B) or codon 200 (C) were analyzed by pyrosequencing. Observed frequencies (mean ± SD) were plotted against input TAC frequencies in (A) and (C) and input GCA frequencies in (B). Regression plots (including linear equation and Pearson correlation coefficient) with 95% confidence bands are shown.

**Figure 3:**
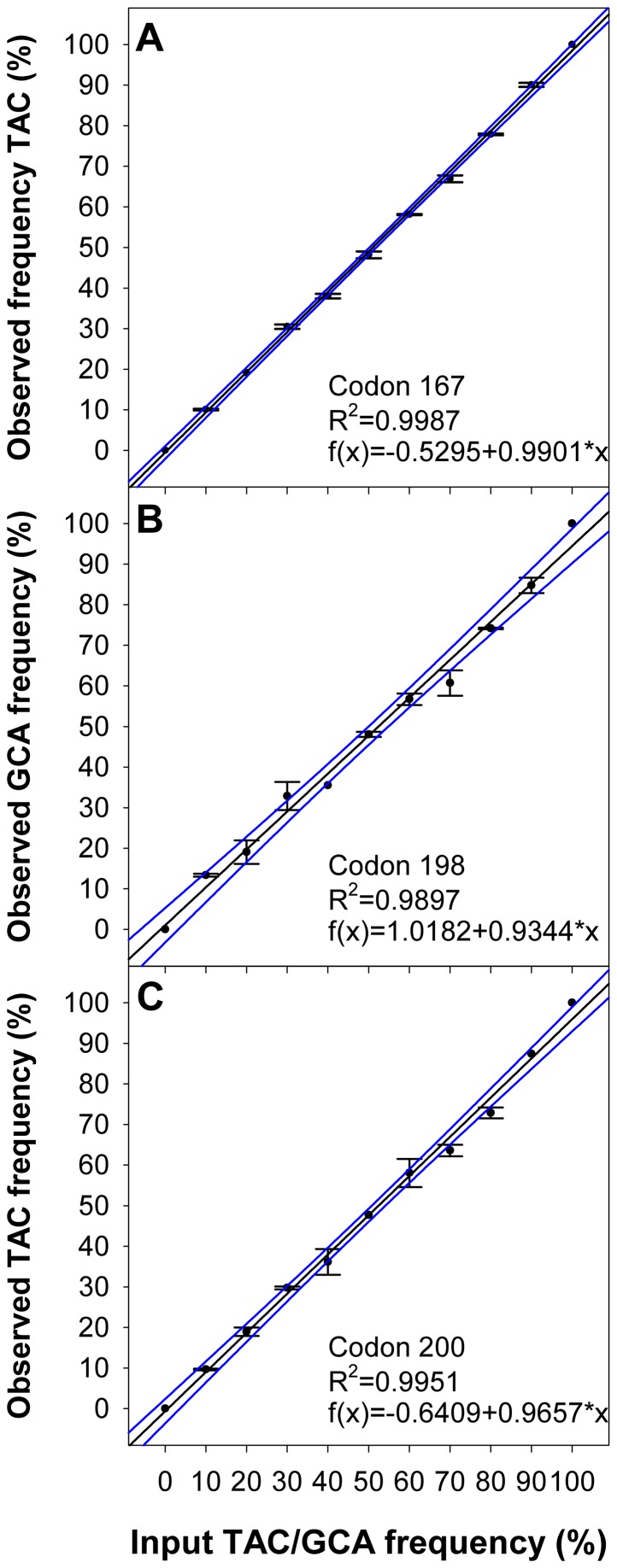
Regression analysis for pyrosequencing assays of *O. ostertagi* β-tubulin isotype 1. Artificial mixtures of respective PCR-products as templates containing varying amounts of copies with TAC in codon 168 (A), codon 198 (B) or codon 200 (C) were analyzed by pyrosequencing. Observed frequencies (mean ± SD) were plotted against input TAC frequencies in (A) and (C) and input GCA frequencies in (B). Regression plots (including linear equation and Pearson correlation coefficient) with 95% confidence bands are shown.

To determine potential BZ resistance associated polymorphisms in the β-tubulin genes of the cattle nematodes, a pyrosequencing assay was developed. Through *in vitro* mutagenesis artificial PCR fragments of β-tubulin isotype 1 of *C. oncophora* and *O. ostertagi* were created which contained the alleles associated with susceptible and resistant phenotypes in other nematode species. Employing these constructs dilution series of the resistant and the susceptible alleles with the following ratios: 10∶0, 9∶1, 8∶2, 7∶3, 6∶4, 5∶5, 4∶6, 3∶7, 2∶8, 1∶9 and 0∶10 were prepared. For the analysis of the SNP frequencies in codon 167 as well as codons 198 and 200 respective sequencing primers were employed (Table S3). An excellent correlation (R^2^ = 0.99) of calculated and observed frequencies for the SNP determination of codon 167, 198 and 200 in the β-tubulin-1 was obtained for both, *C. oncophora* and *O. ostertagi* (see Figures 2 and 3).

Cross-reactivity was excluded by running the species specific assays (*Ostertagia*, *Cooperia* und *Haemonchus*) with the respective heterologous controls (*Ostertagia*, *Cooperia*, *Haemonchus*, *Trichostrongylus* and *Teladorsagia*).

### Resistance status of analyzed isolates and populations

For five of the investigated populations FECRT data was obtained. In Columbia and Argentina calves infected with a field population were divided into two groups and either subjected to ivermectin (both countries, Col.ML & Arg-ML), albendazole (Columbia, Col-BZ) or fenbendazole (Argentina, Arg-BZ) treatment. For the Columbian populations FECRs were 72% (95% confidence interval 37–90%) for ivermectin and 37% (95% CI 0–67%) for albendazole. In Argentina, FECRs were 76% (95% CI 46–93%) for ivermectin and 53% (95% CI 0–85%) for fenbendazole.

In Germany, the Ger-BZ population was obtained from a farm where a FECRT using ivermectin (FECR 81%, 95% CI 71–95%) revealed reduced ML-efficacy. A second FECRT was performed using albendazole, resulting in 70% (95% CI 28–89%) reduction after treatment. Species specific PCR identified *Cooperia* and *Ostertagia* pre and post treatment.

The *O.o.*BZ-sel isolate currently tolerates a subtherapeutic dose up to 40% albendazole (40%: FECR 94%; 35%: FECR 68%). Higher dosages have not been applied yet. In order to further validate the resistance status of this isolate and also due to the absence of FECRT data for the Australian isolate *O.o.*Hamilton2010, EHAs were performed using the method previously published by [37]. The obtained EC_50_ values were 0.113 (95% CI 0.108–0.118) for the *O.o.*Hamilton2010 and 0.064 (95% CI 0.060–0.069) for the *O.o.*BZ-sel. isolate, both significantly (p<0.0001) higher than those of susceptible isolates with EC_50_ values of 0.037 (95% CI 0.036–0.038) and 0.043 (0.042–0.045) for *Ostertagia* and *Cooperia*, respectively. The results of *in vivo* (FECRT) and *in vitro* analyses (EHA) are summarized in Table S5.

### Allele frequency analyses of laboratory isolates and field populations

**Figure 4:**
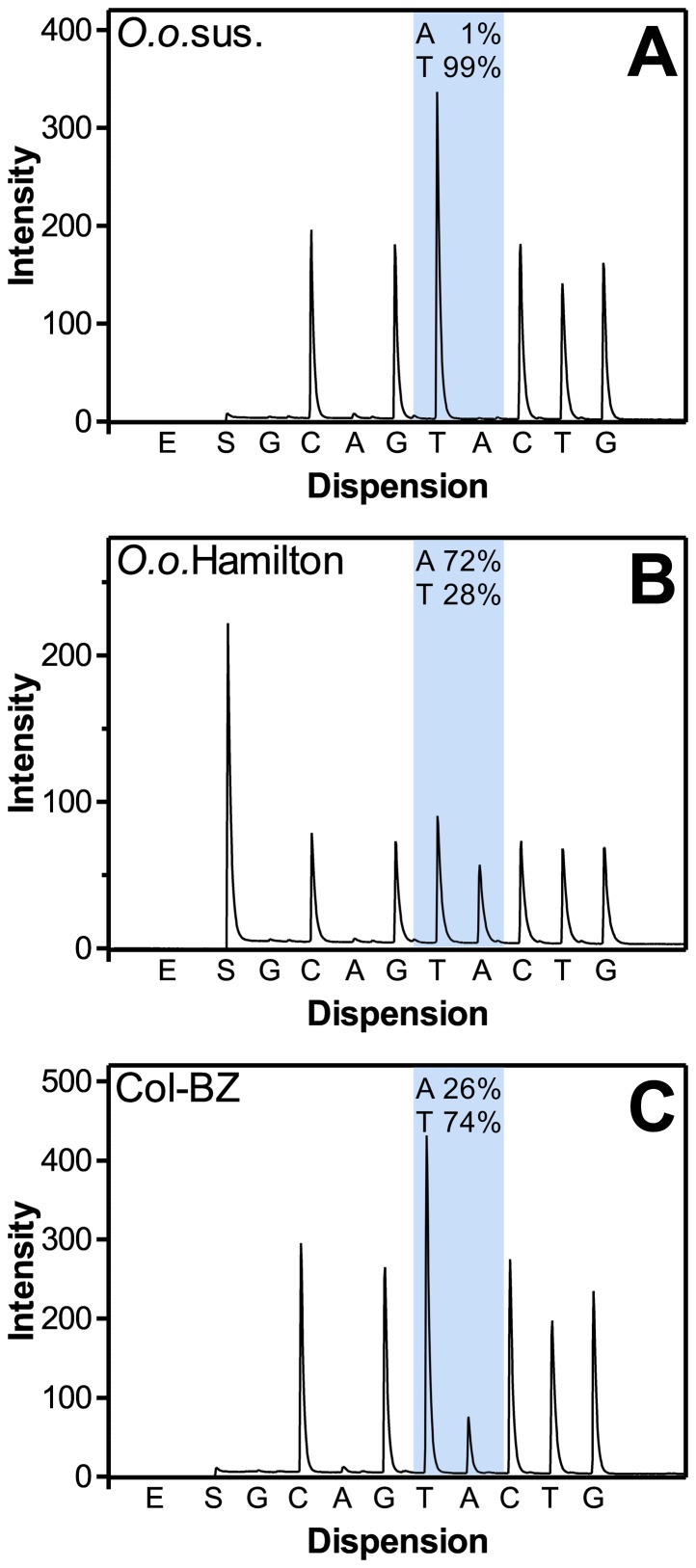
Representative pyrograms for *Cooperia oncophora* and *Ostertagia ostertagi* β-tubulin isotype 1 SNPs. Pyrograms show signal intensity on the ordinate and the dispersion pattern on the ordinate. Dispersions are abbreviated as follows: E, enzyme; S, substrate, A, dATP, C, dCTP, G, dGTP, T, dTTP.

In total, three pure species laboratory isolates (*C.o.*sus., *O.o.*sus. and *O.o.*-BZ-sel.) and six field isolates (Arg-ML, Arg-BZ, Col-ML, Col-BZ, *O.o.*Hamilton2010, Ger-BZ) containing multiple species were analyzed. All field isolates were subjected to both, *Cooperia* and *Ostertagia* specific assay while the susceptible laboratory isolates were only used for the respective species assay. Representative pyrograms for *O. ostertagi* codon 200 are shown in Figure 4. For both susceptible isolates, the frequencies of the alleles correlating with susceptibility were very high (>97%) with the exception of the *O. ostertagi* codon 198 (94%). All field populations were either *Ostertagia* and *Cooperia* (*O.o.*Hamilton2010 >95% *Ostertagia*, Ger-BZ >90% *Cooperia*) or mixed species infections with at least *Ostertagia* sp., *Cooperia* sp., *Haemonchus* sp. (Col-ML, Col-BZ, Arg-ML and Arg-BZ) and additionally very few *Trichostrongylus* sp. (Col-ML and Col-BZ).

While only very few changes in allele frequency were obtained in the *Cooperia* populations (Table 1), a variety of different SNPs present in different combinations was found for *Ostertagia* populations (Table 2). In *Cooperia* SNPs in codon 198 and 200 were significantly increased only in Columbian populations and only after BZ treatment when compared with the susceptible reference isolate or the survivors following ML treatment (p<0.001). The SNP present in *Ostertagia* β-tubulin-1 in codon 167 was the most prevalent (six out of seven populations), followed by codon 200 (five out of seven populations) and codon 198 (two out of seven populations). Between the analyzed field populations no consistent pattern of selection at a particular codon was observed. Again, significantly increasing allele frequencies after BZ treatment (compared to ML treatment) were observed for the Columbian populations, with p<0.001 in codon 167 and p = 0.027 in codon 200. No changes occurred following different treatments in the SNP present in codon 198 (p = 0.744). For the Argentinian population a similar pattern was observed, resulting in a significant increase of allele frequency in codons 167 & 200 (p<0.0001) following BZ treatment and no significant increase in codon 198 (p = 0.069).

**Table 1:** Allele frequencies (resistance-associated alleles) of *Cooperia oncophora* β-tubulin isotype 1 at codons 167, 198 and 200.

Isolate	Codon	*p* value^a^	Codon	*p* value^a^	Codon	*p* value^a^
	167		198		200	
	Mean		Mean		Mean	
	SD		SD		SD	
*C.o.*sus.	0.50		2.67		3.67	
	0.58		3.14		1.75	
*O.o.*Hamilton2010	1.25	ns^b^	2.00	ns^b^	4.20	ns^b^
	0.50		2.16		2.17	
Ger-BZ	0.00	ns^b^	1.00	ns^b^	4.00	ns^b^
	0.00		0.82		1.73	
Col-ML	0.00	ns^b^	0.75	ns^b^	4.25	ns^b^
	0.00		0.98		3.78	
Col-BZ	1.25	ns^b^	30.00	<0.001	18.40	<0.001
	0.50		4.32		4.56	
Arg-ML	0.00	ns^b^	0.33	ns^b^	0.50	ns^b^
	0.00		0.58		0.58	
Arg-BZ	0.00	ns^b^	0.33	ns^b^	1.25	ns^b^
	0.00		0.58		0.96	

a Comparison of different isolates/populations with the susceptible reference isolate *C.o.*sus. using ANOVA and Dunnett's post hoc test.

b Not significant.

**Table 2:** Allele frequencies (resistance-associated alleles) of *Ostertagia ostertagi* β-tubulin isotype 1 at codons 167, 198 and 200.

Isolate	Codon	*p* value^a^	Codon	*p* value^a^	Codon	*p* value^a^
	167		198		200	
	Mean		Mean		Mean	
	SD		SD		SD	
*O.o.*sus	3.40		6.6		1.25	
	0.89		4.39		0.50	
*O.o.*BZ-sel.	69.25	<0.001	6.25	ns^b^	20.40	<0.001
	0.96		0.96		0.89	
*O.o.*Hamilton2010	8.75	<0.05	6.60	ns^b^	73.80	<0.001
	1.50		2.51		4.82	
Ger-BZ	88.20	<0.001	9.00	ns^b^	1.00	ns^b^
	4.21		0.82		0.00	
Col-ML	12.75	<0.001	12.00	<0.01	20.50	<0.001
	3.59		1.41		3.79	
Col-BZ	77.00	<0.001	11.67	<0.05	30.67	<0.001
	2.45		1.16		5.03	
Arg-ML	2.25	ns^b^	6.25	ns^b^	3.20	ns^b^
	0.50		0.96		1.64	
Arg-BZ	22.00	<0.001	4.75	ns^b^	17.33	<0.001
	0.82		0.96		1.16	

a Comparison of different isolates/populations with the susceptible reference isolate *O.o.*sus. using ANOVA and Dunnett's post hoc test.

b Not significant.

Significantly increased frequencies of the resistance associated alleles varied between 8.75 and 88.2% (Tables 1 and 2). Remarkably, in two of the populations all three SNPs were significantly increased in frequency. In further three populations, including the only *Cooperia* population with increased SNP frequencies, at least two SNPs were detected.

Due to very limited amounts of material only the field sample from Columbia after BZ treatment could be analyzed for the presence of SNPs in *Haemonchus* spp. and only codons 167 and 200 were analyzed. The resistance associated alleles were present at frequencies of 13.50%±0.58% and 67.25%±1.71%, respectively. A second SNP a few bases downstream (third position in codon 169) was used to determine whether this population was *H. contortus* or *H. placei*. This revealed that 80.5%±2.38% of the population consisted of *H. contortus*.

## Discussion

In the last decade an increasing number of reports on resistance in cattle nematodes have been published [4], [5], [31], [38]–[44]. Several reasons have been considered as explanation for the so far comparatively limited prevalence of anthelmintic resistance in cattle nematodes, for instance different breeding systems and less intensive anthelmintic use [45]. These recent developments demonstrate the need to find suitable methods for early detection of anthelmintic resistance in the field which can be used in strategies aiming at the prevention or at least postponement of further resistance development [30]. This is especially relevant for the case of cattle trichostrongylids and their susceptibility against BZs, since recent European field studies found that this drug class apparently still shows excellent efficacy [43]. This is in contrast to the respective situation in small ruminants or horses where this drug class has lost its efficacy to a great degree in nearly all regions of the world [3], [7]. Accordingly, a cost efficient and reliable BZ-resistance test for cattle trichostrongylids would be of major benefit for the industry in order to delay onset and spread of resistance.

Pyrosequencing has been shown to be a powerful method for detection of SNPs associated with resistance for several species in different phyla [46]–[48]. For the detection of BZ resistance associated SNPs in parasitic nematodes, pyrosequencing has been previously described for a sheep nematode species by [30], where the codons 167, 198 and 200 of β-tubulin isotype 1 and 2 of different BZ resistant and susceptible isolates of *H. contortus* were successfully examined for genetic variations. Recently the method was first applied for the detection of BZ resistance associated SNPs in the human parasitic nematodes *A. lumbricoides* and *T. trichiura*, which can help to adapt helminth control programs to prevent a development of clinically relevant BZ resistance in human worms [18]. However, the fact that there are three closely related β-tubulins in *A. suum* implies that reliable monitoring of populations by pyrosequencing will need identification of all paralogs that might be potentially involved in BZ resistance and development of pyrosequencing assays for all of them.

Indeed, the phylogenetic analysis presented here indicates that what is called a β-tubulin isotype 1 or isotype 2 is not the same in different nematode lineages and that careful selection of candidate genes where BZ-mediated selection might occur is necessary. The resolution of the phylogenetic relationship in particular between the closely related β-tubulins was not possible on protein level. On cDNA level, however, most alignment programs, including ClustalW2, MUSCLE, T-Coffee and Dialign, were unable to produce useful alignments (data not shown). Only the combination of alignment on protein level followed by maximum likelihood analysis on the aligned codons (cDNA level) provided sufficient resolution.

Although a re-designation of nematode tubulins and in particular β-tubulins according to the rules recently proposed by Beech et al. [49] would be highly desirable, there are a few arguments to postpone this. Firstly, the complex phylogenetic relationship between the large number of tubulin paralogs found in different nematode species apparently complicates a stringent renaming of these genes. Future progress in the diverse, currently running nematode genome and transcriptome sequencing projects will provide data for a more comprehensive phylogenetic analysis of nematode tubulins. This should include the complete repertoire of different paralogs from several nematode species belonging to different clades. Secondly, applying the rules to trichostrongylids β-tubulins isotype 1 and 2 would result in the names ben-1.1 and ben-1.2, respectively. Since both, isotype 1 and isotype 2 [50] have been implicated in BZ resistance the name ben-1.2 would probably be the best choice. However, phylogenetic analysis recently published by Saunders et al. [51] proposed a closer relationship between *Caenorhabditis* ben-1 and *Caenorhabditis* β-tubulin isotype 1 and 2 than to the β-tubulins of trichostrongylids. These authors detected high heterogeneity at the third codon position and therefore eliminated this position from the alignment, suspecting potential homoplasy. The fact that our analysis groups together all β-tubulin genes which are known to be involved in BZ resistance in clade V nematodes appears to be an argument that the phylogenetic hypothesis presented herein reflects the correct evolutionary pathways. Thirdly, nothing is known about the BZ susceptibility of mutants in β-tubulins other than ben-1. The fact that increases in frequency of TAC in codon 200 of a tbb-4/mec-7 (*C. elegans, C. briggsae*) like *T. trichiura* β-tubulin-1 [18] have been reported, suggests that alterations in BZ susceptibility might also correlate with mutations in other β-tubulins. However, there is apparently only one β-tubulin gene encoded in the genomes of both, *T. trichiura* and *T. spiralis*. This suggests that this β-tubulin has to take over all functions which are split amongst different paralogs in other nematodes. Therefore it would probably be best to rename ben-1 and use tbb-5 instead, which is currently listed as a synonym for ben-1 in Wormbase. Finally, it would be highly desirable to seek for consensus between parasitologists and the *C. elegans* community before re-designation of the tubulins to obtain a widely accepted, informative and long-lasting solution.

SNPs in β-tubulin genes of several nematodes were shown to be linked with BZ resistance. While the β-tubulin isotype 1 of *C. oncophora* had been described previously [24], herein we aimed to complete the identification of the most interesting tubulin genes in *C. oncophora* and *O. ostertagi*. Full length sequences of β-tubulin isotype 1 and 2 of *O. ostertagi* and α-tubulin of both species were amplified. At the time of these experiments no BZ resistant *C. oncophora* and *O. ostertagi* populations were available for analysis. To facilitate the development of a molecular test for resistance on basis of the detection of SNPs in the β-tubulin gene, we artificially generated PCR fragments containing SNPs in the codons 167, 198 and 200, cloned them and mixed them to different amounts of “resistant” and “susceptible alleles”. Subsequently, a pyrosequencing assay was established to detect different quantities of “resistant alleles” and to provide a practical tool to determine the resistance status of a single animal or a population. A very good consistency in the calculated and observed allele frequencies in all three codons for both species was obtained. Thus the assay allows the detection of even small frequencies of resistance associated alleles in a larval population which gives the opportunity for early detection of resistance and timely changes within the anthelmintic management procedures before resistance establishes.

Resistance against BZs was previously shown to be associated with a TTC/TAC polymorphism in codon 200 of β-tubulin isotype 1 in field isolates of *C. oncophora* and *O. ostertagi* [24], [25]. In the current study seven field populations resistant to BZ and also MLs were subjected to SNP analysis. These field populations comprised mixed species of which the majority were *Cooperia* and *Ostertagia* and in the South-American populations also *Haemonchus*. In contrast to the results published previously [24], [25], SNPs were also found in the codons 167 and 198 in *Ostertagia* and in codon 198 in *Cooperia*. In the latter, the SNP in codon 198 was only found in the Columbian population after BZ treatment (Col-BZ) and was seen to be significantly increased (p<0.0001). In *C. oncophora* from Columbia, it was surprisingly the SNP with the highest frequency (30%) and additionally combined with a significant (p<0.0001) increase also in codon 200 (allele frequency 18.4%). In only two *Ostertagia* populations an increase of the resistance associated allele in codon 198 was found in comparison to the susceptible reference isolate. In both cases the frequency was comparably low (11–12%) and the increase was only significant in one case (p = 0.031 and p = 0.096). Further analyses are required in order to substantiate, that such low allele frequencies contribute to BZ resistance. It is noteworthy that particularly in those populations resistance associated SNPs in at least one of the other codons are present in much higher frequencies.

In *O. ostertagi*, the most prevalent resistance associated SNP was identified in codon 167 (six out of seven populations), which is in marked contrast to results previously published for *H. contortus* [30], [52]. Allele frequencies were 4.3% for the susceptible isolate and varied between 8.75–88.2% for the six resistant field populations. The fact that in several populations allele frequencies correlating with resistance were simultaneously increased for different codons, might explain why BZ resistance can arise in susceptible populations rather fast.

Both samples analyzed from Columbia and Argentina were paired, i.e. animals from the same herd were allocated to two different treatment groups. Therefore, populations collected after ML and BZ treatment were derived from the same parental population. In both cases allele frequencies in codons 167 and 200 in *Ostertagia* significantly increased after BZ treatment (p<0.0001 for the Columbian populations and p = 0.027 for the Argentinian population) while differences in codon 198 were not observed for *Ostertagia* (p = 0.744 and p = 0.067, respectively). This clearly indicates that BZ treatment selected for the resistance associated alleles in codon 167 and 200 but apparently not in codon 198. Therefore, the weakly increased frequency in codon 198 is probably not responsible for any BZ resistant phenotype.

In contrast to *Ostertagia*, BZ treatment of the Columbian population significantly increased the frequency of the resistance associated SNPs in codon 198 and 200 (P<0.0001 for codon 198 and 0.0013 for codon 200) without changes in codon 167 in *Cooperia*. Although the number of resistant populations analyzed in the present study is still fairly small, selection was observed at all three codons. This suggests that in both species resistance can occur due to mutations arising independently in any of the three codons.

Studies on other parasitic nematodes such as cyathostomins [12], [53]–[55] and human soil-transmitted helminths [18] showed varying degrees of BZ resistance associated selection of the above mentioned β-tubulin SNPs, whereas generally in small ruminant nematode species the mutation in codon 200 was the most frequent one. Noteworthy, recent studies in *H. contortus* revealed interesting associations between the selection towards ML resistance and the above mentioned β-tubulin polymorphisms [17] suggesting that ML selection could also result in selection of BZ resistance associated SNPs. This further adds to the importance of monitoring any potential changes in allele frequencies of these polymorphisms.

In earlier studies directed at sheep nematodes, the pyrosequencing method has been compared to conventional *in vitro* methods such as EHA and other molecular tests such as real-time PCR and was found to correspond with the data obtained by *in vitro* testing and to be time-saving as well as more sensitive [28], [30]. The data presented here show that pyrosequencing is well suited to detect resistance in mixed species cattle nematode infections in the field. For the *O.o.*Hamilton2010 isolate, an EC_50_ well above the cut-off value of 0.1 µg/ml TBZ (originally established for various small ruminant nematode species) and for the BZ-selected *O.o.*BZ-sel. isolate an EC_50_ close to this threshold were obtained. The latter isolate would clearly be diagnosed as susceptible when applying a standard FECRT using the full therapeutic dosage of BZ. The selected isolate, derived from the susceptible reference isolate used in the present study, is under selection for BZ resistance and repeated exposure to BZs did not lead to a resistant phenotype according to data from FECRT using the full therapeutic dose yet. Decreased susceptibility can only be demonstrated using subtherapeutic doses, in this case 35%. However, borderline resistance was detected in the EHA using the standard protocol and the molecular assay provided clear proof of selection. In this case a significant increase of BZ resistance associated SNPs was obtained after subtherapeutic BZ treatments indicating that pyrosequencing is able to detect selection for BZ resistance already in an early stage and might therefore be more sensitive than *in vivo* (FECRT) and maybe also *in vitro* (EHA) methods. Surprisingly, the increase of the codon 167 allele was relatively high (69.25%) while codon 200 was only moderately increased (20.40%). This is in contrast to the results obtained for the fully BZ resistant *O.o.*Hamilton2010 isolate, where codon 200 was strongly (73.80%) and codon 167 only weekly (8.75%) increased. The Col-BZ population shows an *Ostertagia* genotype comparable to the *O.o.*Bz-sel. isolate but much stronger BZ resistance in the FECRT, which might be influenced by the presence of resistant *Cooperia*. These results underline the fact, that selection occurs at all three codons and correlation of resistant phenotype and genotype is rather complex. This is particularly true for interpretation of mixed species infections, where discrimination of species is very difficult in the FECRT requiring larval cultures. In addition, other factors contributing to BZ resistance such as Pgps or cytochrome P450s cannot be excluded. A larger number of samples from systematic surveys including all three techniques would be required to compare the sensitivity of those methods to reliably detect BZ resistance.

## Conclusions

Compared with the more frequent occurrence of cattle nematode populations showing resistance against MLs, the current situation for the BZs appears not that severe at least in Europe [43]. However, it has to be considered that due to a growing resistance problem for MLs, BZs may be again applied more frequently in the future. Accordingly, the risk of increasing BZ resistance will probably rise. This problem can already be seen in countries such as Columbia and Argentina, where ML resistance is widespread and BZs are currently losing their efficacy too.

The presented study provides additional insight in the phylogeny of β- and α-tubulins of the cattle parasites *C. oncophora* and *O. ostertagi*. Furthermore a pyrosequencing assay for the analysis of the frequency of anthelmintic resistance associated β-tubulin SNPs at the codons 167, 198 and 200 of the isotype 1 gene of *C. oncophora* and *O. ostertagi* was developed. This molecular method provides a fast, sensitive and reliable tool for the *in vitro* assessment of BZ-susceptibility on cattle farms, which by the use of pooled samples can be offered for limited costs and thus may be of immediate practical advantage in the field. Evaluation using field populations demonstrated the relevance of the same SNPs previously identified in small ruminants to be also of importance of parasites of cattle.

## Supporting Information

Figure S1 **Enlarged phylogram showing β-tubulin of nematodes and** ***D. melanogaster*** **.** Enlargement of the β-tubulin subtree shown in Figure 1. Statistical support according to the Shimodaira-Hasegawa modification of the approximate likelihood test and the Bayesian transformation of the approximate likelihood ratio are displayed before and after the slash. If only one number is given the results of both tests were identical. Species abbreviations: *Aca*, *Ancylostoma caninum*; *Alu*, *Ascaris lumbricoides*; *Asu*, *Ascaris suum*; *Bma*, *Brugia malayi*; *Can*, *Cylicocylus nassatus*; *Cbr*, *Caenorhabditis briggsae*; *Cca*, *Cylicocyclus catenatus*; *Cel*, *Caenorhabditis elegans*; *Con*, *Cooperia oncophora*; *Cpe*, *Cooperia pectinata*; *Dme*, *Drosophila melanogaster*; *Hco*, *Haemonchus contortus*; *Oos*, *Ostertagia ostertagia*; *Peq*, *Parascaris equorum*; *Tsp*, *Trichinella spiralis*; *Ttr*, *Trichuris trichiura*.
(PDF)

Table S1 **Abbreviation and accession numbers of nucleotide GenBank entries used for phylogenetic analysis.**
(XLS)

Table S2 **Primer sequences for amplification of ß-tubulin gene fragments of** ***Cooperia oncophora*** **(Co) and** ***Ostertagia ostertagi*** **(Oo) for** ***in vitro*** **mutagenesis.**
(PDF)

Table S3 **Primer sequences applied in the pyrosequencing assay.**
(PDF)

Table S4 **Analyzed sequences and programmed dispersions of dNTPs.**
(PDF)

Table S5 ***In vitro*** **and** ***in vivo*** **analyses results for the laboratory isolates and field populations.**
(PDF)
