# Supplementary figures and images for: Phylogenetic Characterization of β-Tubulins and Development of Pyrosequencing Assays for Benzimidazole Resistance in Cattle Nematodes
Source: PLoS One. 2013 Aug 12;8(8):e70212. doi: 10.1371/journal.pone.0070212 (PMC3741318; doi:10.1371/journal.pone.0070212)

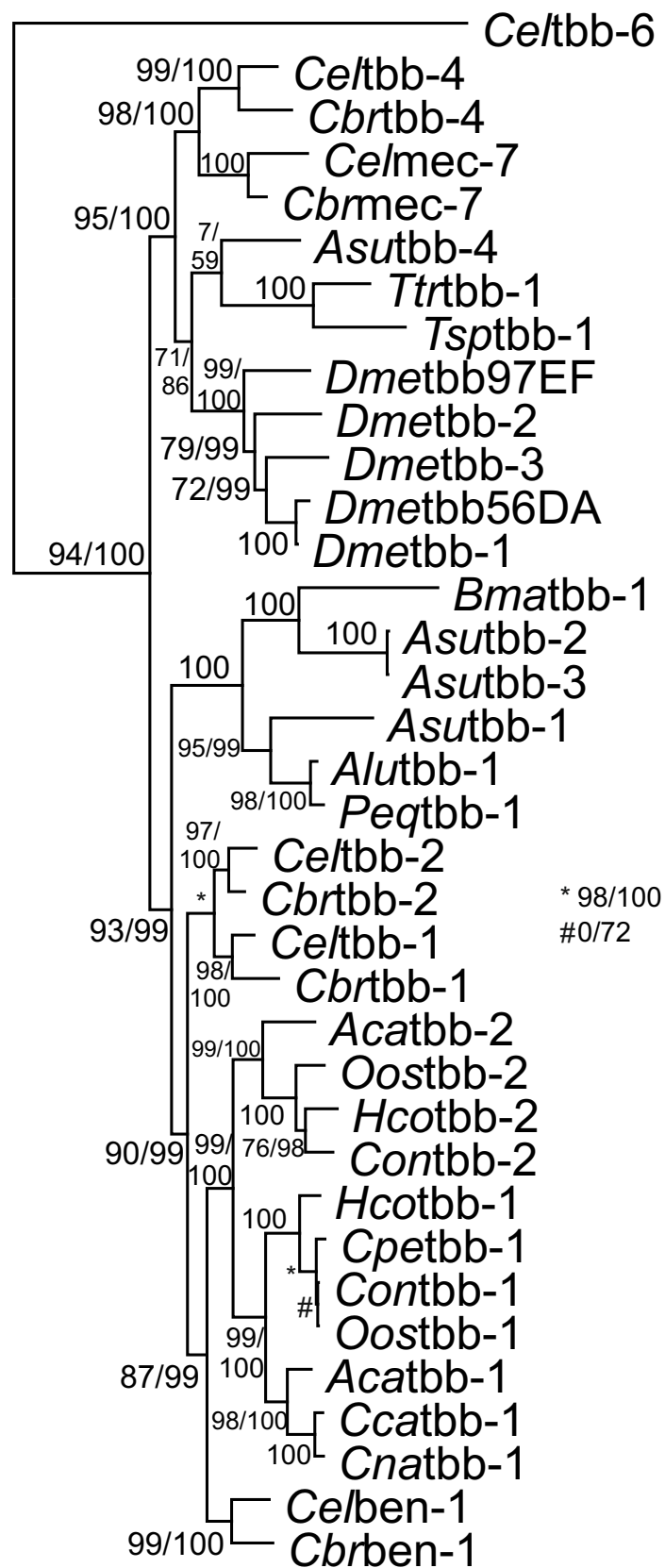

Supplement: Figure S1 — Enlarged phylogram showing β-tubulin of nematodes and D. melanogaster . Enlargement of the β-tubulin subtree shown in Figure 1. Statistical support according to the Shimodaira-Hasegawa modification of the approximate likelihood test and the Bayesian transformation of the approximate likelihood ratio are displayed before and after the slash. If only one number is given the results of both tests were identical. Species abbreviations: Aca, Ancylostoma caninum; Alu, Ascaris lumbricoides; Asu, Ascaris suum; Bma, Brugia malayi; Can, Cylicocylus nassatus; Cbr, Caenorhabditis briggsae; Cca, Cylicocyclus catenatus; Cel, Caenorhabditis elegans; Con, Cooperia oncophora; Cpe, Cooperia pectinata; Dme, Drosophila melanogaster; Hco, Haemonchus contortus; Oos, Ostertagia ostertagia; Peq, Parascaris equorum; Tsp, Trichinella spiralis; Ttr, Trichuris trichiura. (PDF) [file pone.0070212.s001.pdf]
