# Supplementary material for: Phylogenetic Characterization of β-Tubulins and Development of Pyrosequencing Assays for Benzimidazole Resistance in Cattle Nematodes
Source: PLoS One. 2013 Aug 12;8(8):e70212. doi: 10.1371/journal.pone.0070212 (PMC3741318; doi:10.1371/journal.pone.0070212)
Supplement: Table S2 — Primer sequences for amplification of ß-tubulin gene fragments of Cooperia oncophora (Co) and Ostertagia ostertagi (Oo) for in vitro mutagenesis. (PDF) [file pone.0070212.s003.pdf]

Table S2. Primer sequences for amplification of ß-tubulin gene fragments of *Cooperia oncophora* (Co) and *Ostertagia ostertagi* (Oo) for *in vitro* mutagenesis.

Primer name	Primer sequence	Fragment name
CoPCR167fw	5'- TAT GGG CAC TTT GCT TAT TTC A-3'	Coß-tbb1-198/200r
Co198/200mut-rev	5'-ACA GTA CGT TGC ATCGGT ATT TTC TAC CAG TTG GGT GAA C-3'	
Co167mut-fw	5'- CGT ACT CTG TTG TTC CTT CAC CAA AGG TCT CCG ACA CC-3'	Coß-tbb1-167r
CoPCR198/200 rev	5'- CCG GAC ATT GTG ACA GAC ACT AGG-3'	
OoPCR167fw	5'-TCG CCA AAA TTC GTG AGG A-3'	Ooß-tbb1-198/200r
Oo198/200mut-rev	5'-CAG TAC GTC GCA TCA GTA TTT TCC ACC AAC TGA TG-3'	
Oo167mut-fw	5'-CAT ACT CCG TTG TTC CTT CGC CCA AGG TCT C-3'	Ooß-tbb1-167r
OoPCR198/200rev	5'-GTT TTA GTG TGC GGA AGC AAA TAT-3'	

The fragments include either the benzimidazole resistance associated allele in codon 167 (ß-tbb1-167r) or in the codons 198 and 200 (ß-tbb1-198/200r).
