# Supplementary material for: Phylogenetic Characterization of β-Tubulins and Development of Pyrosequencing Assays for Benzimidazole Resistance in Cattle Nematodes
Source: PLoS One. 2013 Aug 12;8(8):e70212. doi: 10.1371/journal.pone.0070212 (PMC3741318; doi:10.1371/journal.pone.0070212)
Supplement: Table S3 — Primer sequences applied in the pyrosequencing assay. (PDF) [file pone.0070212.s004.pdf]

Table S3. Primer sequences applied in the pyrosequencing assay.

No	Primer name	Primer sequence	Ta[a]
1	CoPCR167fw	5'-TAT GGG CAC TTT GCT TAT TTC A-3'	62
2	CoPCR167rev+B	5'-Biotin -ACG TTT CAT CGG TAT TTT CTA CCA-3'	
3	CoPCR198+200fw	5'-CGA CAC CGT TGT GGA ACC TTA C-3'	60
4	CoPCR198+200rev+B	5'-Biotin -CCG GAC ATT GTG ACA GAC ACT AGG-3'	
5	OoPCR167fw	5'-TCG CCA AAA TTC GTG AGG A-3'	55
6	OoPCR167rev+B	5'-Biotin -GAG ACC TTG GGC GAA GGA A-3'	
7	OoPCR198+200fw	5'- GTT CCT TCG CCC AAG GTC T-3'	57
8	OoPCR198+200rev+B	5'- Biotin -TGT GCG GAA GCA GAT ATC GTA-3'	
9	Oo200fw	5'- GGT GGA AAA TAC TGA TGA GAC GT-3'	53
10	Oo200rev+B	5'- Biotin -GTT TTA GTG TGC GGA AGC AAA TAT-3'	
11	CoSeq.167	5'-ACA GAA TTA TGG CTT CGT-3'	
12	CoSeq.198	5'-ACT GGT AGA AAA TAC CGA T-3'	
13	CoSeq.200	5'-AAA TAC CGA TGA AAC GT-3'	
14	OoSeq.167	5'- CGG ATA GAA TCA TGG CTT-3'	
15	OoSeq.198	5'- TGG TGG AAA ATA CTG ATG-3'	
16	OoSeq.200	5'- GGT GGA AAA TAC TGA TGA GA-3'	

Primers 1 – 10 were used for PCR, reverse primers carried a biotin tag (+B). The numbers 167, 198 and 200 indicate the benzimidazole resistance associated alleles in the ß-tubulin gene. Primers 11 – 16 were used for the sequencing reaction.

[a] Ta, annealing temperature.
