# Supplementary material for: Phylogenetic Characterization of β-Tubulins and Development of Pyrosequencing Assays for Benzimidazole Resistance in Cattle Nematodes
Source: PLoS One. 2013 Aug 12;8(8):e70212. doi: 10.1371/journal.pone.0070212 (PMC3741318; doi:10.1371/journal.pone.0070212)
Supplement: Table S4 — Analyzed sequences and programmed dispersions of dNTPs. (PDF) [file pone.0070212.s005.pdf]

Table S4. Analyzed sequences and programmed dispersions of dNTPs

Assay	Codon	Sequence to analyze	Dispersion order
H.c. Codon167	167	**W**CTCCGTTGTT	GATCTCGT
H.c./p. Codon167	167	**W**CTCCGT<u>Y</u>GTT	GATCTCAGTCG
H.c.=p. Codon200	200	**W**CTGTATTGACAACGAAG	GATCTGTA
C.o. Codon167	167	**W**CTCTGTTGTTCCTTCAC	GATCTCTG
C.o. Codon 200	200	**W**CTGTATTGATAATGA	GATCTGTA
C.o. Codon 198	198	G**M**AACGTWCTGT	TGCACAGTACTG
O.o. Codon 167	167	CAT**W**CTCCGTT	TCGATACTC
O.o. Codon 198	198	**M**GACGTWCTGTA	GACGACAGTACTG
O.o. Codon 200	200	CGT**W**CTGTATCGAT	GCAGTACTG

Species abbreviations: *H.c.*, *Haemonchus contortus*; *H.p.*, *Haemonchus placei*; *C.o. Cooperia oncophora*; *O.o.*, *Ostertagia ostertagia*
SNPs used for genotyping of β-tubulin alleles are shown in bold, the SNP used for discrimination of *H. placei* and *H. contortus* is underlined.
