# Supplementary material for: Phylogenetic Characterization of β-Tubulins and Development of Pyrosequencing Assays for Benzimidazole Resistance in Cattle Nematodes
Source: PLoS One. 2013 Aug 12;8(8):e70212. doi: 10.1371/journal.pone.0070212 (PMC3741318; doi:10.1371/journal.pone.0070212)
Supplement: Table S5 — In vitro and in vivo analyses results for the laboratory isolates and field populations. (PDF) [file pone.0070212.s006.pdf]

Table S5. *In vitro* and *in vivo* analyses results for the laboratory isolates and field populations

Isolate/Population	Treatment	FECR[a] (95% CI[b])	EHA[c] EC_{50} (95% CI[b])
*C.o.*sus	Albendazole	100%	0.043 (0.042-0.045)
*O.o.*Hamilton2010	n.a.[d]	n.a.[d]	0.113 (0.108-0.118)
Ger-BZ	Ivermectin	81% (71-95%)	n.a.[d]
	Albendazole	70% (28-89%)	
Col-ML	Ivermectin	72% (37-90%)	n.a.[d]
Col-BZ	Albendazole	37% (0-67%)	n.a.[d]
Arg-ML	Ivermectin	76% (46-93%)	n.a.[d]
Arg-BZ	Fenbendazole	53% (0-85%)	n.a.[d]
*O.o.*sus	Albendazole	100%	0.037 (0.036-0.038)
*O.o.*BZ-sel.	Albendazole 40%	94%[e]	0.064 (0.060-0.069)
	Albendazole 35%	68%[e]	

[a]Fecal Egg Count Reduction Test
[b]Confidence Interval
[c]Egg Hatch Assay
[d]Not available
[e]CI not available as only 2 animals used per group
